# Optometrists' Perspectives Regarding Artificial Intelligence Aids and Contributing Retinal Images to a Repository: Web-Based Interview Study

**DOI:** 10.2196/40887

**Published:** 2023-05-25

**Authors:** Aurora Constantin, Malcolm Atkinson, Miguel Oscar Bernabeu, Fiona Buckmaster, Baljean Dhillon, Alice McTrusty, Niall Strang, Robin Williams

**Affiliations:** 1 School of Informatics University of Edinburgh Edinburgh United Kingdom; 2 Centre for Medical Informatics Usher Institute University of Edinburgh Edinburgh United Kingdom; 3 Centre for Clinical Brain Sciences University of Edinburgh Edinburgh United Kingdom; 4 Department of Vision Sciences Glasgow Caledonian University Glasgow United Kingdom; 5 School of Social and Political Science University of Edinburgh Edinburgh United Kingdom

**Keywords:** AI in optometry, repository of ocular images, user studies, AI decision support tools, perspectives of optometrists and ophthalmologists, AI, research, medical, decision support, tool, digital tool, digital

## Abstract

**Background:**

A repository of retinal images for research is being established in Scotland. It will permit researchers to validate, tune, and refine artificial intelligence (AI) decision-support algorithms to accelerate safe deployment in Scottish optometry and beyond. Research demonstrates the potential of AI systems in optometry and ophthalmology, though they are not yet widely adopted.

**Objective:**

In this study, 18 optometrists were interviewed to (1) identify their expectations and concerns about the national image research repository and their use of AI decision support and (2) gather their suggestions for improving eye health care. The goal was to clarify attitudes among optometrists delivering primary eye care with respect to contributing their patients’ images and to using AI assistance. These attitudes are less well studied in primary care contexts. Five ophthalmologists were interviewed to discover their interactions with optometrists.

**Methods:**

Between March and August 2021, 23 semistructured interviews were conducted online lasting for 30-60 minutes. Transcribed and pseudonymized recordings were analyzed using thematic analysis.

**Results:**

All optometrists supported contributing retinal images to form an extensive and long-running research repository. Our main findings are summarized as follows. Optometrists were willing to share images of their patients’ eyes but expressed concern about technical difficulties, lack of standardization, and the effort involved. Those interviewed thought that sharing digital images would improve collaboration between optometrists and ophthalmologists, for example, during referral to secondary health care. Optometrists welcomed an expanded primary care role in diagnosis and management of diseases by exploiting new technologies and anticipated significant health benefits. Optometrists welcomed AI assistance but insisted that it should not reduce their role and responsibilities.

**Conclusions:**

Our investigation focusing on optometrists is novel because most similar studies on AI assistance were performed in hospital settings. Our findings are consistent with those of studies with professionals in ophthalmology and other medical disciplines: showing near universal willingness to use AI to improve health care, alongside concerns over training, costs, responsibilities, skill retention, data sharing, and disruptions to professional practices. Our study on optometrists’ willingness to contribute images to a research repository introduces a new aspect; they hope that a digital image sharing infrastructure will facilitate service integration.

## Introduction

Community optometrists in Scotland are being asked to contribute their collections of retinal images to a National Health Service (NHS) repository to enable research focusing on the earlier stages of eye diseases. This should enable improvements in the detection and treatment of those conditions. Optometrists are the first port of call for people with an eye problem as “optometrists (as graduates) are trained to examine the eyes to detect defects in vision, signs of injury, ocular diseases or abnormality and problems with general health, such as high blood pressure or diabetes. They make a health assessment, offer clinical advice, prescribe spectacles or contact lenses, and refer patients for further treatment, when necessary” [1]. We asked optometrists for their thoughts about contributing their patients’ data and their expectations about potential benefits and challenges.

Clinical research on diagnosis and treatment of eye diseases is largely confined to hospital ophthalmology services and universities. This is problematic; for example, clinical trial recruitment fails to reach individuals whose eye conditions (eg, dry age-related macular degeneration) fall outside a referable disease threshold. To overcome this barrier, the Scottish Collaborative Optometry-Ophthalmology Network e-research (SCONe) seeks to create a repository of retinal images captured by optometrists in the community [2]. This will facilitate new clinical research. SCONe will also be an educational resource for auditing false-positive and false-negative referrals and provide exemplars, variants, and outliers. The former will improve patients’ pathways between primary and secondary care. The latter will improve clinical image interpretation. SCONe’s image repository will enable carefully governed research spanning the full diversity of Scottish patients and all stages of disorders. Images will be gathered from optometry practices, with 2 substantial benefits: (1) a nearly complete coverage of the population attending primary care optometry services and (2) coverage of a broad spectrum of disease severities, including early and undiagnosed disease as well as those with no disease. Information about diagnosis, treatments, and outcomes will depend on pseudonymized linkage to standard health care data sets.

The advent of the SCONe repository and the need to *build back better* (a UK rallying cry after COVID-19) motivated our study. We focused on optometrists because they are in the front line of eye health care, and in Scotland, they are provided extra training and NHS-provided cameras. Nearly 900 optometry practices employ 1300 optometrists in Scotland (Table 1). Optometrists refer 3%-9% of their patients to hospitals, with 89%-97% accuracy in Scotland [3]. Their wide distribution makes them more accessible to patients than hospital services. Optometrists’ collaboration with ophthalmologists is crucial. Therefore, we interviewed a small sample of ophthalmologists as well as optometrists to better understand their working relationship with optometrists.

**Table 1:** Characteristics of Scottish optometry practices [4].

Practice type	Practices (n)	Description of practice
Domiciliary	76	business providing eye care in a patient’s home or care setting
Independent	473	an individual or small group of locally owned optometry practices
Multiple	299	part of a large (typically national) chain or franchise of practices, for example, Specsavers or Boots Opticians

SCONe will improve artificial intelligence (AI) methods by exploiting the contributed images. The unprecedented population diversity and coverage will permit data-driven training and validation that potentially addresses recently highlighted issues of bias in AI methods. The images represent patients’ histories preceding recognized onset of eye conditions. They may therefore contain latent information that would have enabled earlier diagnoses. Such early predictors would be significant in several of the eye conditions identified by Campbell et al [5], where AI has the potential to improve eye care. The validation against the full population addresses one impediment to the uptake of AI-enabled methods identified by González-Gonzalo et al [6]. They propose a multi-step strategy, whereby carefully chosen sets of relevant stakeholders are fully engaged through all 7 stages—from planning to operation. Both these papers draw attention to the difficulty of moving from research to practical widespread use. Our interviewees’ perceptions of that challenge are a significant element of González-Gonzalo et al’s [6] first stage.

Given the aging population and the growing numbers and range of conditions that can now be treated, optometrists, as the eye-care front line, need help—potentially from AI. Our study focuses on optometrists to better understand their needs and constraints as they consider contributing patients’ images to a shared repository and prepare to use AI-powered assistance in their expanding primary eye care role. Although optometry services in Scotland have some particularities [7], our findings can be generalized to most countries.

## Methods

### Overview

 This study employs semistructured interviews to identify the attitudes to changes in the use of retinal images among practicing optometrists and ophthalmologists, particularly with respect to changes stimulated by SCONe. We conducted 23 web-based interviews between March and August 2021 via Microsoft Teams. Each interview lasted for 30-60 minutes. Only the interviewee and 1 interviewer (the first author) were present, except for the first ophthalmologist, while 2 other authors attended to refining the script for ophthalmologists.

The interview script was designed to reveal the expectations, issues, and constraints encountered by optometrists, for example, their uses of the shared images and worries over extra work and training that contributing images might require. The interview scripts were revised in consultation with our expert advisors and after 2 pilot interviews retaining topical consistency, as per a previous study report [8]. The questions covered 4 categories: (1) image sharing, (2) AI-enabled methods, (3) research, and (4) education/training related to image sharing and AI.

We also conducted a limited set of interviews with ophthalmologists to explore the crucial optometry-ophthalmology collaboration—primarily during referral but also during training and when negotiating revisions of responsibilities. The script for ophthalmologists was revised drawing on experience from 8 optometrist interviews retaining their topics and adding 2 new topics: (5) opening questions and (6) ophthalmology-optometry relationships (as per a previous study) [8].

We interviewed 23 people (18 optometrists of which 9 were females and 5 ophthalmologists of which 4 were females). Initially, we recruited interviewees through SCONe and extended and diversified our sample through snowball techniques and other channels. We covered a representative sample of optometrists that included both smaller independent practices and larger multiple practices (eg, national chains). The interviews included 14 optometrists from independent practices and 4 optometrists from multiple practices, with coverage of the diversity of practice contexts shown in Table 2. Recruitment was slow because interviewees had excessive workloads handling the pandemic backlog, which, given our completion deadline, restricted the number and range of recruits. However, toward the end, interviews revealed very few new issues; so, we believe we have validated our scripts and methods and can provide a good representation of optometrists’ views. For the ophthalmologists, we tried to cover various specialisms, for example, age-related macular degeneration, diabetic retinopathy, and glaucoma.

**Table 2:** Optometry interview coverage of Scottish urban/rural categories [9] showing ratios of interviewees in each category compared with the population sizes and number of optometry practices

Category	Optometrists (n=18), n (%)	Population size (n=5,454,000), n (%)	Optometry practices (n=900), n (%)
Large urban area	12 (66.6)	1,887,000 (34.6)	337 (37.5)
Other urban area	3 (16.6)	1,974,000 (36.2)	394 (43.8)
Accessible small town	1 (5.6)	464,000 (8.5)	72 (8)
Remote small town	1 (5.6)	191,000 (3.5)	60 (6.7)
Accessible rural area	1 (5.6)	611,000 (11.2)	13 (1.5)
Remote rural area	0 (0)	322,000 (5.9)	24 (2.6)

The interviews (total of 15 hours) were recorded and professionally transcribed, introducing pseudonyms to protect identities but permit follow-up studies. The analysis followed the thematic analysis method [10] using NVivo (Lumivero) [11]. Thematic analysis is one of the most widely used methods in qualitative studies. Its purpose is to identify themes (ie, patterns) in the data and relationships between themes that are relevant to a specific research topic/phenomenon. Thematic analysis is well-suited for analyzing large texts such as transcripts of a set of interviews. The next section presents the themes developed from our data with the corresponding evidence (quotes) that supports them. Quotes are associated with a pseudonym P number, allocated when someone agreed to be interviewed. The characteristics of those interviewed are tabulated in [8]. Quotes from ophthalmologists are discriminated by “ophthalmologist” following a participant’s ID; all other quotes are from optometrists.

### Ethics Approval

This study has been approved by the School of Informatics (University of Edinburgh) Research Ethics Committee (RT 62378).

## Results

### Overview

The interviews and analysis revealed consistent commitment to maintaining high professional standards and improving eye care by using new methods and technologies notwithstanding worries about costs and workloads. Five themes came out from the analysis. Their order results from ordering topics in the scripts are as follows: (1) changes to professional working patterns, (2) envisaging the image repository’s impact, (3) benefits from AI decision support, (4) paths to improved eye care, and (5) education and training. We present our analysis structured by these themes summarizing significant views expressed, with the number of interviewees who supported each point, out of 18 for optometrists and 5 for ophthalmologists. We present extracts of the transcripts retaining the abbreviations they used.

### Theme 1: Changes to Professional Working Patterns

Funding pressures were a predominant issue as optometrists considered increasing their clinical responsibilities. Optometrists and ophthalmologists drew attention to the commercial pressures on optometrists in their competitive market of small private enterprises and larger multiples where prescribing spectacle frames and lenses is financially as well as clinically essential (12 optometrists, 3 ophthalmologists).

…I think sometimes optometry is strange because it is…90% clinical but there is a commercial aspect to it…competition with each other.

P3

Those from independent practices felt they had more choice over their allocation of effort.

…Independent practices work very differently …I don’t feel time pressured, …if I need more time, I take more time …. the majority of optometrists, especially [those in] multiples, do not [have that luxury].

P8

The divergence between prescribing lenses and diagnosing other eye conditions may be reinforced by patient attitudes.

…people who don’t believe that there’s a problem with their eyes just want to go and get glasses.

P28

Five optometrists expressed concerns over meeting the costs.

…the way the GOS [General Ophthalmic Services] contract is structured clinical stuff isn’t the thing that pays the bills. So, something that’s going to generate workload, but not potentially generate [income] is going to be a tough sell.

P2

These choices are affected by public policy and funding. P24 explained that “The contract … in Scotland empowers us more, pays us more, pays us to [monitor] conditions.” P25 noted that although they are doing more tests and interpretation of the results and spending time explaining these to patients, “the NHS fee hasn’t changed very much at all.” The initial fee reflected the cost, but it has not been increased in line with inflation and additional procedures. Optometrists in Scotland take more responsibilities in health care [7].

…I would say that that is less common in Scotland now [to have optometrists who do not want to do more than prescribe lenses] because we’ve been doing this sort of work …for a long time now. …There’s a budget for training and developing optometrists.

P24

To reduce the burden on secondary care and to obtain good quality images, one of the ophthalmologists proposed the establishment of specialist imaging hubs.

…It would be very useful to have imaging hubs where imaging equipment can be standardized and similar to those used in NHS Ophthalmology Departments…high resolution photographs which are essential for safe management of patients. The resolution we want is virtually impossible for all the optometric practices to have.

P22, ophthalmologist

Eleven optometrists and 4 ophthalmologists proposed to expand the role of optometrists.

…Hopefully, it will enable us to provide a better service for patients. There might be times where you do not have to refer to hospitals and you will manage someone locally.

P15

One optometrist believes “a huge number of optometrists are willing to take on more responsibility” but suggested that this could stimulate stratification of the profession.

…We need to start having some kind of differentiation in the hierarchy in eye care. Not just optometrists and ophthalmologists but a continuum between the two.

P28

This expansion of roles should be carefully analyzed (as indicated by 4 optometrists and 2 ophthalmologists). For example, extra skills will be needed. However, optometrists may not receive additional remuneration.

…The idea behind it is that we’re moving more professionally and we’re moving into a better, more rewarding profession, but we’re not having enough money.

P25

### Theme 2: Envisaging the Image Repository’s Impact

All optometrists were keen to see an extensive and long-running research repository containing their patients’ images. Its primary role is to improve AI. However, its educational role and coordination may facilitate communication and collaboration. The following issues dominated: (1) professional relationships, (2) teleconsultations, (3) health inequalities, (4) image quality, (5) standardization and automation, (6) scrutiny out of context, and (7) the need for electronic health records (EHRs). Interviewees expanded our vision of what mattered, which was a goal of our in-depth interviews. They highlighted the critical needs.

Most optometrists (12/18, 67%) hoped the image repository would facilitate supporting each other.

…We can learn from other people’s treatment management plans…we can enhance a collective learning and collective management of patients.

P18

Optometrists (10/18, 56%) hoped the shared repository would stimulate a change in relationships between optometrists and ophthalmologists.

…A project like this could…make optometrists feel more part of the whole…linking primary and acute services so that you’re out in the community, but we are part of the hospital project and yes, we engage with this data gathering which suddenly gives you the message…your work is appreciated and valued.

P7

Two optometrists hoped that it will mean hospital staff gain increased respect for optometry.

…The communication [between the optometrists and ophthalmologists] would necessarily have to increase …hospital eye care needs to understand the importance of the role of the community care …making their life easier.

P28

This contrasts with ophthalmologists’ conviction that their “relationship with optometrists is very good” [P19, ophthalmologist]. Ophthalmologists were aware of plans to deploy an EHR system—a better pathway to using images when coordinating patient care.

…We’re supposed to be going to a digital, an electronic patient record with images, so if that was shared with optometrists, they might be able to learn from that.

P23, ophthalmologist

Four local [12] optometrists and all the ophthalmologists in this study proposed using teleconsultation, possibly stimulated by its use during the COVID-19 pandemic [13].

…I could see it being really useful for…to bring eye health screening to more remote locations.

P28

…But the benefits are at least timeously we can be getting in touch with the patient and either having a chat with them or reviewing images just to make sure that there is no gross alteration.

P22, ophthalmologist

Teleconsultations should result in more accurate triage.

…I think the benefits of it would definitely be reducing the number of patients who need to be seen in the hospital…bring the images to the doctor instead of the whole patient.

P23, ophthalmologist

One ophthalmologist pointed out the risks of virtual examinations.

…The risk, which I always explain to patients, in a virtual appointment is that it is easy to miss subtle changes in retinal pathology.

P22, ophthalmologist

Three optometrists suggested that data sharing would reduce variations in the care.

…It might be useful, because in some areas there might be more healthy patients, and in others there might be unhealthier patients. The retinal images might help get…understanding…managed more.

P4

Five optometrists were concerned about the quality of the retinal images they produced. Causes included device quality, opacities (eg, cataract), and no dilation. In hospital, dilation is routine. In optometry practices, dilation is the only standard for patients aged over 60 years.

…And I think there’s also a difference, and I don’t know if this has really been considered, between imaging captured in hospital and imaging captured in practice, in that a lot of imaging captured in practice isn’t dilated, isn’t of the quality that is captured in a hospital or a screening service setting.

P2

Three optometrists anticipated that the image repository would raise image quality concerns.

…I think just…standardizing things across the board so the people who are taking the images know.

P10

Six optometrists and 3 ophthalmologists mentioned standardization for images and software applications that “talk to each other.”

…There might be a technology issue and a standard…there are so many different types of devices…different manufacturers…software…many different systems that are independent, is going to be a challenge.

P16

Optometrists anticipated service improvements from image sharing. Two optometrists hoped image sharing would avoid redundant work and improve patient care.

…There’s far too much duplication of services…if we have a centralized service, and all this data is collected, it can only improve for patients…and the health of their eyes.

P2

Four optometrists hoped for more accurate information by accessing patients’ records.

…So…rather than depending on a patient’s word of mouth…patients are not the best in relaying accurate information about their past treatments…it would be nice to be able to access the actual data.

P18

Three optometrists expected the repository would help them track patients’ data.

…Patients move and patients’ care providers move, and patients often attend optometry where they work as opposed to where they live.

P2

Some optometrists feared that an image repository would be used to investigate whether their decisions were right, “putting themselves at undue risk for litigation whenever their records are being pulled apart by other people” [P4].

Another optometrist worried about clinical decisions being scrutinized.

…So, I would be a little bit concerned that… someone would be looking over my shoulder and deciding whether I had made the right decision or not, whereas they didn’t have all the data that I had available.

P28

Eight optometrists worried about the technical requirements and time needed to contribute images.

…That’s the biggest challenge, it’s always…how do we get these images…transferred easily, and that’s not too time consuming.

P5

One optometrist wondered whether the contribution process could be automated.

### Theme 3: Benefits From AI Decision Support

Considering the use of AI decision support, all optometrists anticipated benefits and were happy to use it, provided they retained the ultimate responsibility. They expected more treatment options with better guidance and help when they encounter something new. Their issues included (1) diagnostic skill acquisition and retention, (2) divergence in the optometry community, (3) changing relationships with patients, and (4) impatience over the rate of deployment. The AI tools were seen as augmenting their skills, empowering them to make better decisions (as stated by 14 optometrists).

…I would feel it was an affirmation probably of what I was doing, or even a confirmation of what I was doing.

P5

Ten optometrists felt strongly that they should retain control and that clinical decisions must remain their responsibility.

…You have to take the human factors into account and base it on all your observations not just what the technology is telling you.

P15

Ophthalmologists considered that AI decision support could help optometrists make better referrals and detect eye diseases earlier.

…I have no doubt…[that AI tools help optometrists make better referrals]. It will help a lot … in early diagnosis…one of our main cornerstones in management of glaucoma.

P27, ophthalmologist

More efficient interpretation of the growing number of images may be delivered.

…One of the big things now is with ever-increasing imaging you’ve got an ever-increasing burden of reviewing…If AI allows you to do that at a more efficient pace and a more accurate pace.

P2

Six optometrists expressed concerns that colleagues using these tools might fail to develop critical skills.

…If it’s brought in too early in somebody’s training as a clinician, then [they may] fail to develop their own clinical decision-making skills.

P18

There was a similar concern that skills might fade among those who become too dependent on the new technology, coupled with a risk of misinterpreting “results in the AI.”

…We’d have to be very clear that whoever’s [using] the AI understands how to interpret them, as well as preventing skill fade from clinicians who are used to interpreting these images.

P2

All optometrists but 1 were enthusiastic about taking on new responsibilities to improve patient care. This would require additional skills and professional development. P6 observed that it might create a rift in their community.

…There are some practitioners … a small minority (who typically qualified many years ago) who feel … they were trained to examine eyes and provide optical corrections and they don’t like this whole shift.

P6

Five optometrists feared that reliance on AI assistance would impinge upon their skills and professional judgement and their personal contact with patients.

…There is a risk of reducing the respect, qualifications, and the ability of the optometrist…[AI] is used to replace parts of a test rather than aid.

P10

However, many optometrists considered that new technologies will improve their reputation (7/18) and help them be seen as up-to-date (1/18).

…If you’re explaining to a patient that you’re using AI, they would be very impressed … happy … their optometrist is using up-to-date methods.

P4

…So, I think all those things …. Gives the practice a … standing within the medical community, which would help.

P3

There is a considerable delay from the moment AI research demonstrates a new technique to its wide application. One optometrist expected that the national repository will “reduce this time of ‘translating’ research into practice to 10 years” (compared with 17 years [6]) and “hopefully save some eyesight for people” [P3].

### Theme 4: Paths to Improved Eye Care

Taking a long view of improvements in eye care made possible by the image repository and AI, optometrists expected early detection (18/18), increasing accuracy (10/18), higher efficiency (4/18), better disease progress monitoring (8/18), and risk prediction (7/18). Two research advances using the repository are anticipated: (1) improved AI decision support (tuned for the population and with new predictors) and (2) improved education. These are assumed by optometrists when they discuss long-term benefits.

…We may be able to catch things before they get to a more progressive stage where they are more devastating to sight as well.

P13

Earlier accurate diagnoses would increase optometrists’ efficiency.

…Often, we’ll see patients … for follow-up appointments just because we’re uncertain. But if these AI technologies … even in those grey or uncertain patient situations meant we could make a better or a quicker judgement, then that would be handy.

P18

The speed of diagnosis increases efficiency and reduces patient stress.

…It needs to be as close to real time as possible so that it can be a very clear way of generating a result that doesn’t have the anxiety of waiting on an envelope coming through the door or an email.

P2

Support for managing disease progression is a widely held expectation.

…We need to know whether it’s the same pathology, whether it’s… progressed.

P27, ophthalmologist

Optometrists expect to be in a better position to predict risks.

…Then we would be in a better position to predict patients at risk. Rather than monitor patients who already are showing signs of disease…It may be easier to predict patients at risk of developing certain conditions.

P10

Sustainability was a concern for 6 optometrists. Some suggested that the government should support not just the acquisition of new equipment but also the organizational procedures and systems they needed.

…In Scotland, the funding from Government would have to match the amount of time that’s actually spent using this [AI] equipment to aid with a diagnosis.

P6

### Theme 5: Education and Training

All interviewees agreed that education is needed to prepare them to exploit image sharing and AI tools in a reliable and professional way. The main points revealed were (1) formalizing and incentivizing training, (2) information resources, (3) allocating the time for training, and (4) learning by interacting with ophthalmologists.

Five optometrists suggested developing accredited educational resources about AI and its role in eye care to encourage participation and to validate achievement of standards.

…if all optometrists had to do mandatory training as part of their CPD [Continuing Professional Development]. Part of our mandatory training, then we would all be starting on the same page.

P15

The education of patients and their supporters was perceived by optometrists as crucial. It takes time to discuss new approaches. They needed information that was simple and concise in various formats (eg, on paper, online).

…You’d certainly need something…in terms of cascading the information to patients…an information pack [we] can just hand out.

P6

Steering groups were proposed to raise patients’ awareness of the benefits of AI, discuss concerns, and clarify challenges.

…You could have a steering group with optometrists, ophthalmologists, tech guys, patients represented, to hear their views, but maybe you’re doing that already.

P6

There was an almost universal feeling that the best way to develop the new skills needed was by learning on the job. Several suggestions emerged as interviewees contemplated what might be needed when a small number of ophthalmologists were helping a larger number of optometrists develop their professional expertise. Jointly developed treatment plans were an aspiration for 5 optometrists and 2 ophthalmologists to improve their relationships and train optometrists for more roles. Two ophthalmologists suggested having more optometrists visiting hospitals.

…I’m very [keen to have] hospital optometrists working in our teams. I feel that I can support the training, development and progress of these small cohorts very well rather than communicating with multiple community opticians.

P22, ophthalmologist

## Discussion

Optometrists deliver primary eye care service for the great majority of patients and judge when referral to secondary care is warranted. Our study has established a model for enquiring about their aspirations and concerns. We summarize the wide-ranging discussions, which thematic analysis clustered into 5 themes that interlink.

### Theme 1: Changes to Professional Working Patterns

Changes to the professional working patterns of optometrists are anticipated due to increasing clinical responsibilities and growing workloads. These stresses arise from an aging population with increasingly severe eye conditions and effective treatments extending the duration of care. Extra information provided by an increased number of higher resolution images requires additional interpretation time. Funding was optometrists’ primary concern, with refractive correction a potential source of cross subsidy in prosperous practices. A variety of ways of providing more care in the community anticipated their discussion of theme 4.

### Theme 2: Envisaging the Image Repository’s Impact

Optometrists looked toward (1) professional collaboration, (2) teleconsultations, (3) remedies for health inequalities, (4) image quality issues, (5) effective standards and interworking systems, (6) scrutiny of decisions, and (7) EHR for optometry. The benefits of covering the full diversity of patients and eye conditions predominated. Optometrists’ vision went beyond the direct effects of an image repository. For example, after the pandemic’s restrictions, they envisaged triage improvements from image-sharing teleconsultation. They expected to support colleagues working in deprived communities, and they foresaw an integrated image-handling EHR system improving their management of patients. OpenEyes, being commissioned in Scotland, will meet EHR requirements, but community optometrists will have to wait for its benefits, as it will be deployed initially in hospitals [14]. These extensions and worries over scrutiny reveal misconceptions about the SCONe repository. Its privacy protection extends across all patients and optometrists; so, neither data sharing nor scrutiny are possible within SCONe. When an EHR provides those mechanisms, these opportunities and issues will re-emerge.

### Theme 3: Benefits From AI Decision Support

In this context, optometrists expect (1) more accurate and faster decisions for which they would still take full responsibility, (2) help with conditions not previously encountered, (3) more efficiently interpreting images, (4) short-term status enhancement with patients despite some fears of longer term erosion of expertise and responsibility, and (5) frustration over delays in adopting new methods leading to loss of sight that could have been prevented. Invariably, AI assistance was anticipated positively but with significant concerns about responsibilities, practicalities, and time scales. The support for the image repository and the AI it enables could evaporate unless the pragmatic issues raised regarding system and technical complexities, the impact on already busy workloads, and the navigation of ethical and patient privacy governance are addressed. González-Gonzalo et al [6] recommend a multistep strategy engaging all stakeholders to address this. This careful planning and introduction process is necessary to prepare for provision and to sustain such innovations. There remain uncertainties about the effectiveness in the field of AI-powered decision aids in the context of evolving practices, diversity of equipment, and variations in image-taking procedures inherent in community optometry.

### Theme 4: Paths to Improved Eye Care

Optometrist suggestions included (1) earlier detection of more conditions, (2) increasing triage accuracy, (3) improved efficiency, (4) better condition monitoring, and (5) identifying patients at risk. The earlier detection depends on research enabled by their contribution of earlier images (and similar research) detecting latent signals. Improved triage accuracy depends on the AI assistance and the additional training both enabled by the repository. Studies of whole populations enabling AI assistants to highlight signals they might otherwise miss would underpin these benefits. Interviewees anticipated closer collaboration between primary and secondary care, redistributing responsibilities for diagnosis and treatment for some disorders, leading to new roles for optometrists with additional skill requirements. However, there was concern among optometrists that communication from hospital eye services to optometrists needs improving. An audit highlighted variability (45%-92%) in the successful delivery of formal responses from hospitals to optometrists [3]. Sustainability is a critical issue, involving many more professional roles [6].

### Theme 5: Education and Training

In this context, optometrists proposed (1) incentives via certification, (2) informing patients and supporters, (3) ophthalmology placements, and (4) jointly planned treatments. All interviewees valued training and expected significantly informative extra material drawn from the repository. However, contributing to the repository, using the AI assistants, and taking on extra clinical responsibilities will all require additional training, requiring more resources, time, and materials.

A cross-cutting issue emerged. The difference in perception of the quality of communication between optometrists in the primary care sector and specialist ophthalmologists is worrying and merits further attention. It may result from differences in professional status (eg, reflecting different lengths of qualification path [4 and 7+ years]) as well as the different relationships between professionals and patients (patients are free to switch between optometrists). Improvements in digital communication (EHR) may mitigate or exacerbate this issue. In the evolving primary eye care context revealed by theme 1, themes 2 and 3 meet our objective of discovering optometrists’ attitude to contributing their patients’ retinal images and to using AI assistance. Theme 4 captures their ideas about how eye care may be improved by future service innovations—our second objective. These require training innovations covered by theme 5. As few new ideas emerged in the final interviews, we regard our methodology and evidence gathered as reliable.

Primary eye care in Scotland depends on the skills and diagnostic capabilities of optometrists developed and assessed through the NHS Education and Glasgow Caledonian University [7]. The local culture provides a positive context for these developments. However, we believe that in most other contexts, optometrists would have similar aspirations and concerns. Our methodology and findings should prove beneficial for other countries planning to gather optometry images to improve early detection and triage. Further work should explore these developments in an international context considering all the issues optometrists raised.

### Limitations

As a pilot study, we only interviewed 18 out of 1300 optometrists. Our very small sample has a potential bias that is hard to estimate, as interviewees were recruited through the SCONe project. Therefore, respondents may have been better informed and more inclined to support retinal image contribution. Table 2 shows that we achieved reasonable coverage of geographic diversity.

## Abbreviations

AI: artificial intelligence
EHR: electronic health record
NHS: National Health Service
SCONe: Scottish Collaborative Optometry-Ophthalmology Network e-research
